# Advanced Liquid-Free, Piezoresistive, SOI-Based Pressure Sensors for Measurements in Harsh Environments

**DOI:** 10.3390/s150820305

**Published:** 2015-08-18

**Authors:** Ha-Duong Ngo, Biswaijit Mukhopadhyay, Oswin Ehrmann, Klaus-Dieter Lang

**Affiliations:** 1 Center of Microperipheric Technologies, Fraunhofer Institute IZM, Berlin 13355, Germany; E-Mails: Biswajit.Mukhopadhyay@izm.fraunhofer.de (B.M.); Oswin.Ehrmann@izm.fraunhofer.de (O.E.); kdlang@izm.fraunhofer.de (K.-D.L.); 2 University of Applied Sciences, FB I, Microsystems Engineering, Berlin 12459, Germany

**Keywords:** MEMS, SOI-based sensors, sensors for harsh environment, sensors for high temperature

## Abstract

In this paper we present and discuss two innovative liquid-free SOI sensors for pressure measurements in harsh environments. The sensors are capable of measuring pressures at high temperatures. In both concepts media separation is realized using a steel membrane. The two concepts represent two different strategies for packaging of devices for use in harsh environments and at high temperatures. The first one is a “one-sensor-one-packaging_technology” concept. The second one uses a standard flip-chip bonding technique. The first sensor is a “floating-concept”, capable of measuring pressures at temperatures up to 400 °C (constant load) with an accuracy of 0.25% Full Scale Output (FSO). A push rod (mounted onto the steel membrane) transfers the applied pressure directly to the center-boss membrane of the SOI-chip, which is placed on a ceramic carrier. The chip membrane is realized by Deep Reactive Ion Etching (DRIE or Bosch Process). A novel propertied chip housing employing a sliding sensor chip that is fixed during packaging by mechanical preloading via the push rod is used, thereby avoiding chip movement, and ensuring optimal push rod load transmission. The second sensor can be used up to 350 °C. The SOI chips consists of a beam with an integrated centre-boss with was realized using KOH structuring and DRIE. The SOI chip is not “floating” but bonded by using flip-chip technology. The fabricated SOI sensor chip has a bridge resistance of 3250 Ω. The realized sensor chip has a sensitivity of 18 mV/µm measured using a bridge current of 1 mA.

## 1. Motivation

High accuracy pressure control is a key feature in many industrial processes (e.g., the plastic, ceramic, chemical, aerospace, or pharmaceutical industry) [1,2,3]. However, these processes often require measurements at elevated temperatures (>150 °C). Standard silicon pressure sensors cannot withstand direct exposure to such heat. While many papers on this topic have been published, there are no semiconductor-based sensors for temperature ranges over 250 °C on the market. Measurement via coupling media restricts the dynamics and precision as measured results are subsequently extrapolated. Table 1 compares the known and published technologies used and the performances of the corresponding systems.

**Table 1:** State-of-art pressure sensors for high temperature applications.

Characteristics	Si-Bulk Piezoresistive Non-Compensated	Filled Sensors Available	SiC-Based (not on Market) [4]	SOI-Based (not on Market) [5]	Unit
typical value					
Offset	±7	±7	na *	±10	mV
Span voltage (FSO)	170	170	18–66	120	mV
Sensitivity	8.7 (µV/V kPa)	depends on steel membrane	0.013 (mV/V/psi)	0.29–0.42 (mV/psi)	
TCO	0.01	0.01	−0.18	0.01	%FSO
TCS	0.1	0.1	0.24	0.1	%FSO
Linearity	0.35	0.35	1	0.1–0.5 non compensated	%FSO
Pressure hystresis	<0.1	<0.2	0.7	0.1	%FSO
Temperature range	<250	Oil—300, Mercury—400, NaK—500	600	300	°C
Size	2 × 2 × 0.5	Diameter 7 mm, height 10 mm	na *	aa *	mm^3^

(* not available).

The usage of mercury or oil as a coupling medium (not allowed in the food industry and the EU), the complicated filling technology, contamination of products by the liquids used, and the fact that the steel membrane for media separation is prone to rupture are additional drawbacks. Hence, sensors capable of direct measurement at high temperatures would permit fast, accurate, and simple process control. Pressure sensor chips fabricated from SOI-substrates featuring stand-alone, isolated piezoresistors forming a Wheatstone bridge and a high temperature resistant metallization enable such measurements. Deep silicon etching using a Bosch-process allows small chip size and arbitrary membrane geometry. 

## 2. Sensor Concepts

### 2.1. Floating Sensor Concept

**Figure 1:**
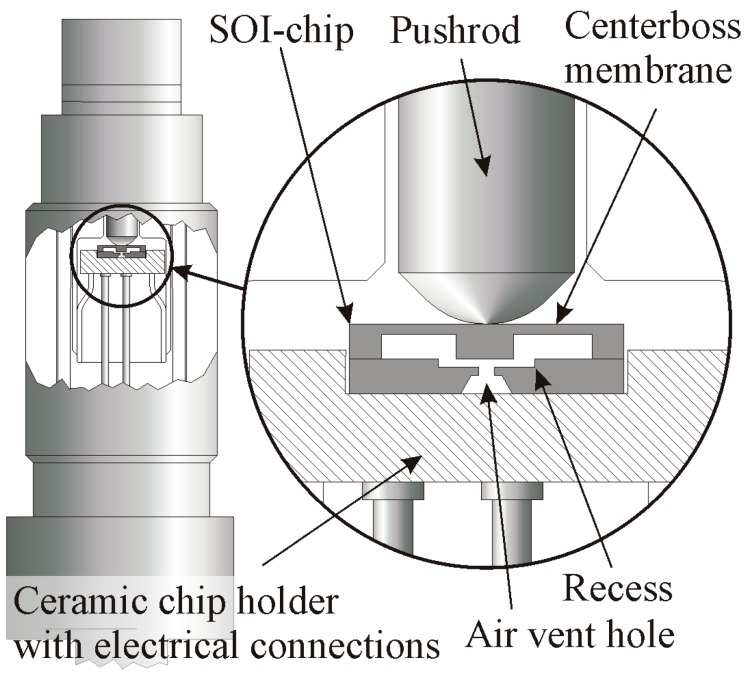
Schematic view on a fully packaged pressure sensor using floating concept, (**left**) with enlarged cross section of the packaged SOI-chip [3,6,7,8].

The sensor concept is shown in Figure 1 [3,6,7]. A ceramic pushrod, connected with the steel membrane by brazing, transfers the steel membrane’s deflection to the SOI sensor chip. The deflection is a function of the applied pressure. The SOI chip is placed into a ceramic holder which provides also the feed through for the electrical contacts. This “floating” sensor concept avoids the use of any additional materials, such as needed in common chip bonding technologies. Additionally, pressure transferring media like Hg, oil or NaK, which are expensive and require complicated filling technologies, are no longer needed. Electrical connections between the SOI chip and ceramic holder are realized by wire bonding. The steel membrane is welded to an outer steel cylinder. The ceramic holder is brazed to an inner Kovar cylinder. Inner and outer steel cylinders are connected by welding.

Materials used for the sensor have been carefully chosen, in order to achieve the best sensor performance at high temperatures and to keep the mechanical stress in SOI sensor element at high temperatures below silicon breakage stress. The thermal expansion coefficients of the Kovar, ceramic and silicon used match best.

**Figure 2:**
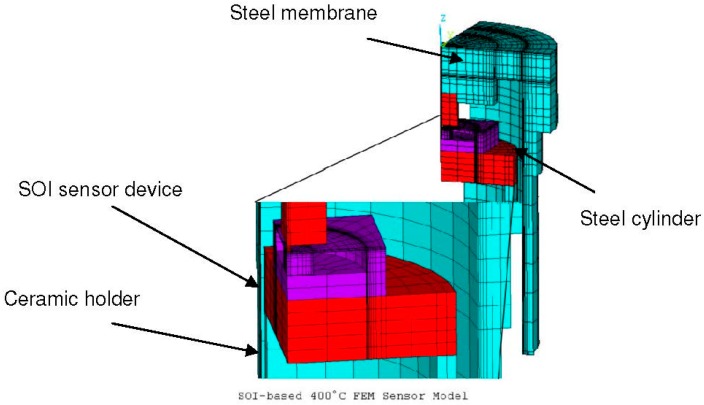
ANSYS FEM model used in this work. **Right**—quarter model of the sensor system, light blue: steel parts, red: ceramic parts, purple: silicon sensor chip which consists of a centerboss membrane, bonded to a silicon wafer; **Left**—enlarged model of the SOI sensor device with centerboss and ceramic holder.

The sensor devices are composed of two silicon wafers connected by direct wafer bonding. The bottom wafer of each chip features a small recess which provides space for the displacement of the centerboss of the SOI-chip. An air venting hole allows pressure balancing. Additionally, the bottom wafer acts as an overpressure safety feature when the centerboss comes in contact with the bottom of the recess (Figure 1). In this work FEM is used to optimize the package and SOI sensor geometries (Figure 2).

During the assembly of the whole sensor, a pre-deflection (of the silicon membrane by the ceramic tip) must be achieved. The physical contact between ceramic tip and silicon membrane must be ensured for the whole temperature range (up to 400 °C). Based on this consideration, the geometries of steel membrane, the ceramic pushrod and pre-deflection can be calculated and optimized. The sensor system has a fixed diameter of 7.8 mm and the outer diameter of the steel membrane of 5.8 mm. The Wheatstone bridge contains four longitudinal piezoresistors in the [110] direction. Two of four piezoresistors are located at the membrane edge and the other two at the centerboss’s edge. This configuration minimizes any stress residue in the silicon membrane caused by different CTEs of the used materials. As all four piezoresistors will face the same stress, this gives the best offset behavior of the sensor.

### 2.2. Flip-Chip-Sensor-Concept

**Figure 3:**
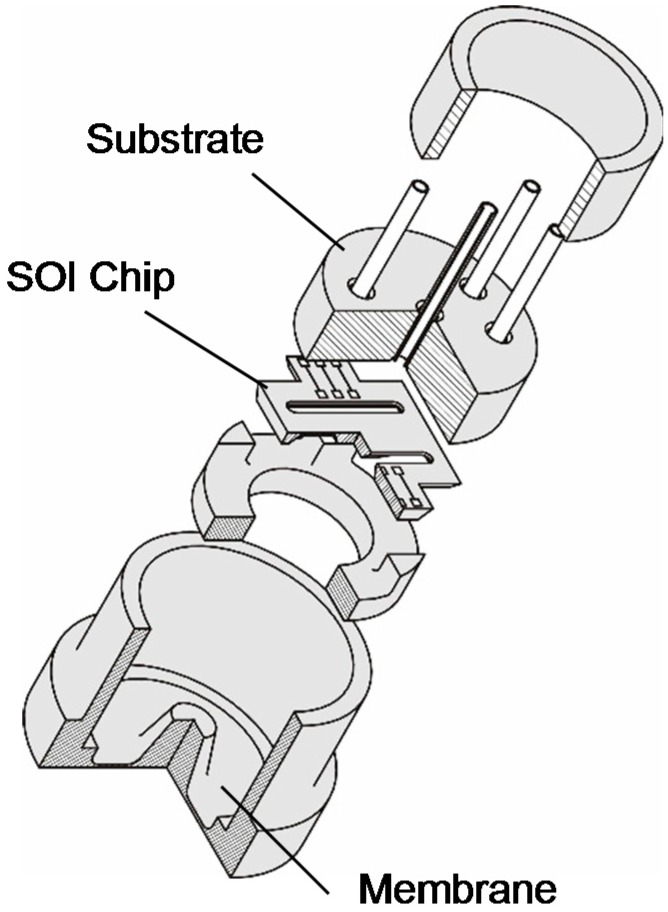
Exploded view of the “Flip-Chip”-Sensor concept for use in harsh environments.

The flip-chip concept used in this work is shown in Figure 3. The steel membrane has a center-boss connected with the SOI chip in the middle. The SOI piezoresistive sensor chip is mechanically and electrically connected with a metallized chip carrier (substrate with feed-through pins for electrical contacts) by using standard flip chip bonding with gold bumps. Gluing for mechanical fixing of the sensor chip is not needed in this concept. The gold bumps are created using a conventional wire bonder with bumping option. The measured pressure acts on a robust steel membrane. The steel membrane’s deflection is transferred onto the silicon chip and can be detected with the integrated piezoresistors. A transferring media such oil is also not needed. The substrate has integrated (feed-through) pins for the supply current and output signal. As bond wires are not needed, this concept is suitable for use in extreme acceleration and high shock applications such as combustion engines. The stiffness of the steel membrane is much larger than that of the sensor chip and so defines the deflection of the sensor system. The sensor chip itself can be considered as a deflection (force) sensor.

## 3. Sensors Fabrication

**Figure 4:**
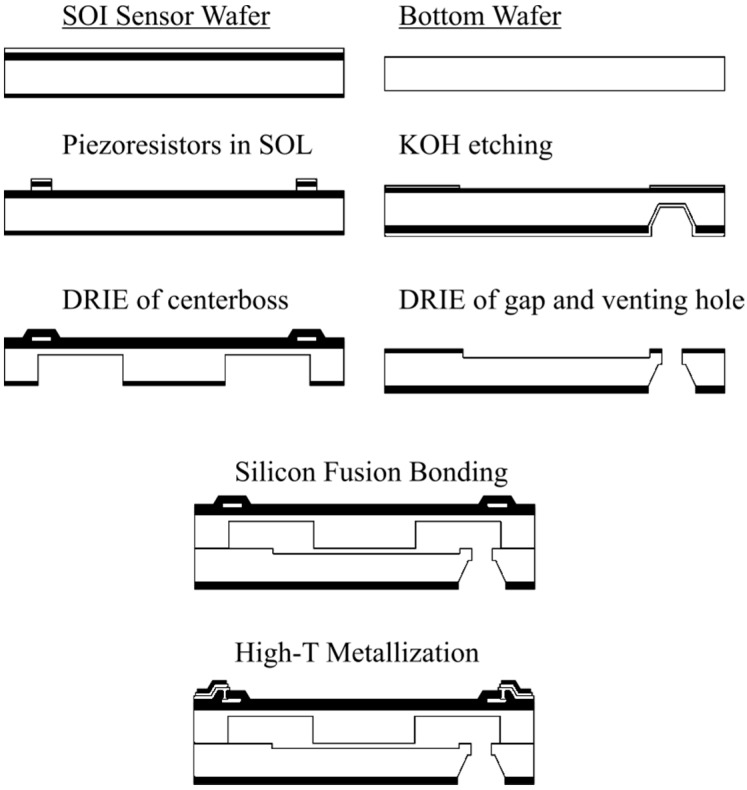
Process flow for sensor fabrication. Piezoresistors and membrane with centerboss are fabricated in SOI wafer. Gap and venting hole are fabricated in Si bottom wafer. SOI and bottom wafers are bonded by using silicon fusion bonding [8].

The fabrication process for the “floating” SOI sensor is shown in the Figure 4. (100) SOI wafer with specific properties from SOITEC (Bernin, France) and silicon wafer from Okmetic (Vantaa, Finland) have been used to produce the sensor element. Two implantation steps and a RIE are needed to create the *p*-piezoresistors and the contact areas. After that a DRIE on the back side of SOI wafer was applied to produce the silicon membrane with centerboss. 

For the bottom wafer, two DRIE steps and a KOH etching were applied to create the gap between centerboss and bottom wafer and the air vent holes. Both wafers were bonded by using direct silicon bonding at 1000 °C. The piezoresistors were passivated and contacted by an appropriate high temperature Ti-based metallization scheme.

**Figure 5:**
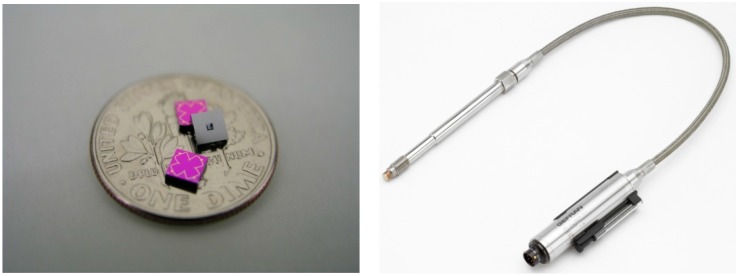
Fabricated SOI chips for floating concept. The chip size is 3.6 × 3.6 mm^2^, compared with one dime coin (**left**). Packaged sensor system for use in plastic injection application (**right**) [8].

**Figure 6:**
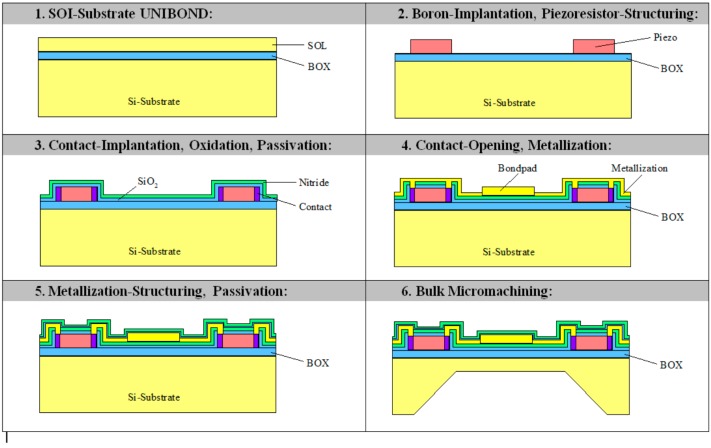
Process flow for sensor fabrication [9].

Figure 5 shows realized “floating” SOI chip. The same SOI material was used for fabrication of the “flip-chip-bonded” SOI sensor. The process flow is shown in Figure 6.

**Figure 7:**
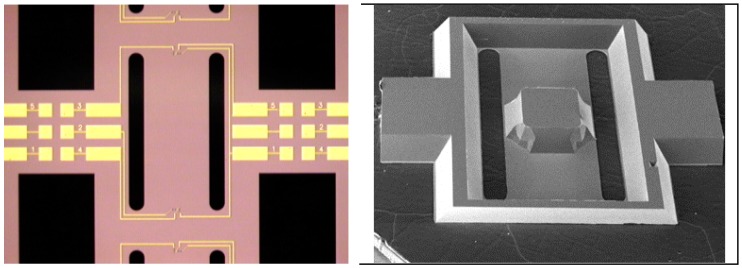
Fabricated SOI sensor chip. **Left**—top view, and **Right**—bottom view of the fabricated device [9].

After implantation and etching of the piezoresistors, they were passivated and electrical contact pads were opened. Next, the high temperature metallization scheme was realized to provide Ohmic contact to the piezoresistors. The silicon sensor beam was realized by combining KOH and DRIE etching. Figure 7 below shows top and bottom views of the fabricated sensor element.

## 4. Test Results

### 4.1. Floating Sensor Concept

**Figure 8:**
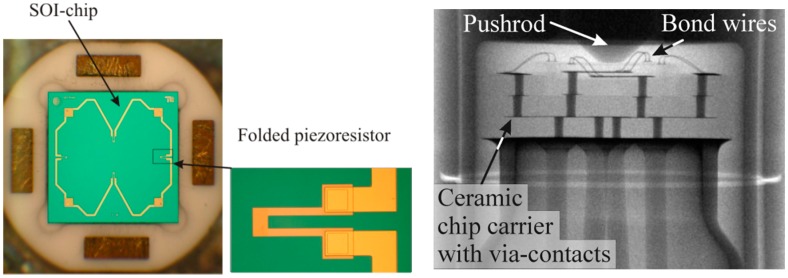
**Left**—top view of SOI-pressure sensor chip placed in ceramic carrier cavity (dotted line indicates centerboss membrane) with detailed view of folded piezoresistor; **Right**—X-ray photograph of the packaged sensor. The bond wires, pushrod and the feed through are visible [8].

Figure 8 (left) shows a picture of a realized pressure sensor chip placed in the ceramic chip carrier cavity and a piezoresistor structure. Figure 8 (right) shows an X-ray photograph of the packaged sensor. The pushrod as well as the ceramic chip carrier with its via-connections can be seen.

Exhaustive testing procedure has been done for more than 1300 h. All sensors have shown very stable and repeatable behavior. No visible damage has been observed. The Wheatstone bridge resistance of the bare SOI-die is 3,7 kOhm at room temperature yielding an average Temperature Coefficient of Resistance (TCR) of 1.3 e^−3^·K^−1^ between RT and 400 °C. 

**Figure 9:**
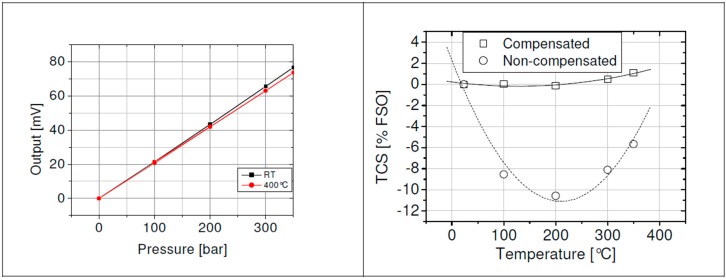
**Left**—Signal output over pressure of the packaged sensor at 25 °C and 400 °C (@ 0.5 mA) for pressure range up to 350 bar; **Right**—TCS (temperature coefficient of sensitivity) of the packaged sensor over temperature. Straight: Compensated package; dotted: Non-compensated package [8].

The output signal over pressure at 25 °C, and 400 °C, respectively, is shown in Figure 9. The end point non-linearity at 400 °C is about 0.2% FSO. 

### 4.2. Flip-Chip-Sensor Concept

**Figure 10:**
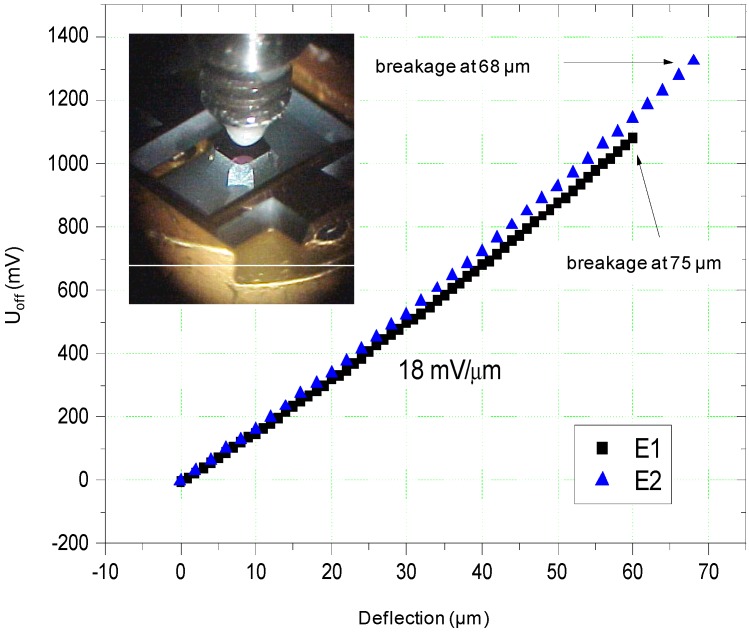
Output signals measured with fabricated SOI sensor chips [9].

The fabricated SOI sensor chip has a bridge resistance of 3250 Ω. The SOI sensor chip was tested with the set up shown in [9]. The realized sensor chip has a sensitivity of 18 mV/µm measured using a bridge current of 1 mA. The measured output signal is presented in Figure 10.

**Figure 11:**
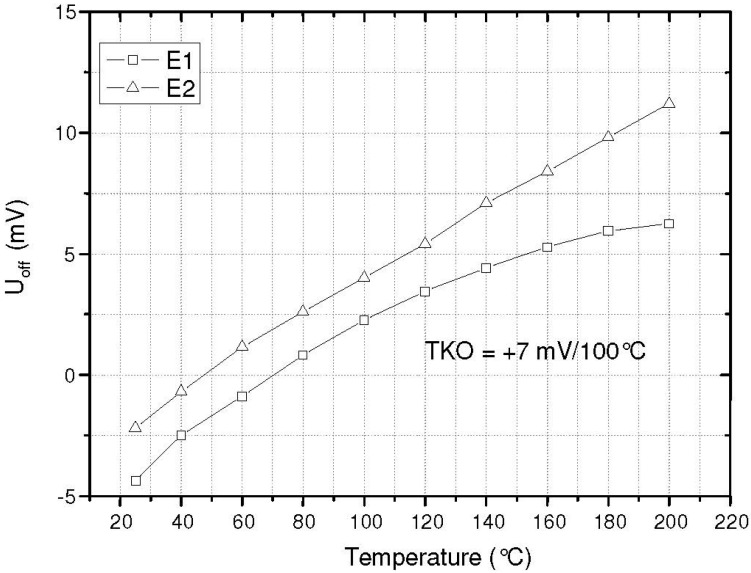
Offset voltage measured with the fabricated sensor [9].

The bridge offset shown in Figure 11 is very low and we can see that the thermally induced stress caused by thermal compression bonding performed at high temperatures is not significant. Thus, the thermal expansion of the chip carrier and the chip are well matched to each other and the decoupling of the specific chip structure is sufficient. This can also be seen at the low TCO (temperature coefficient of the offset) which is about 7 mV/100 K. The linearity error is approx. 1% FSO. 

**Figure 12:**
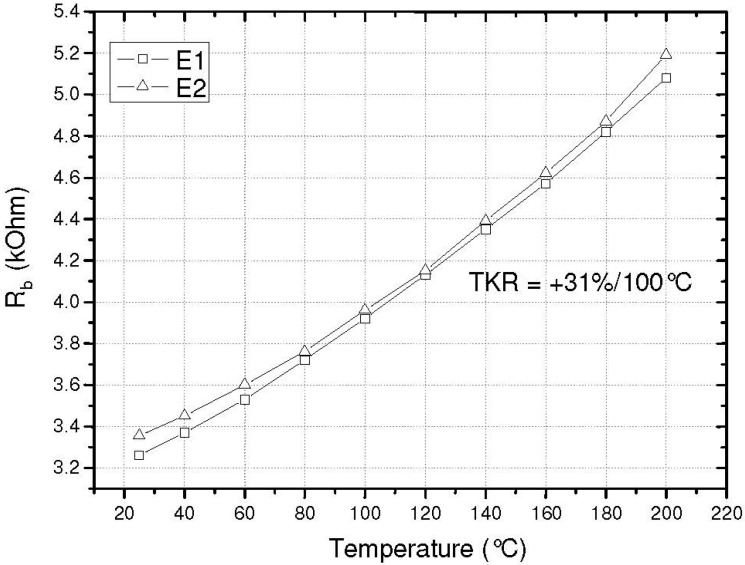
TCR of the SOI sensor chip for temperature up to 200 °C [9].

One requirement of the analog electronics is the positive temperature of the sensitivity (TCS) over the entire temperature range. Thus, the positive TCR has to predominate the negative temperature coefficient of the gauge factor. The TCR of the sensor is 3 × 10^−3^ K^−1^ as shown in Figure 12.

**Figure 13:**
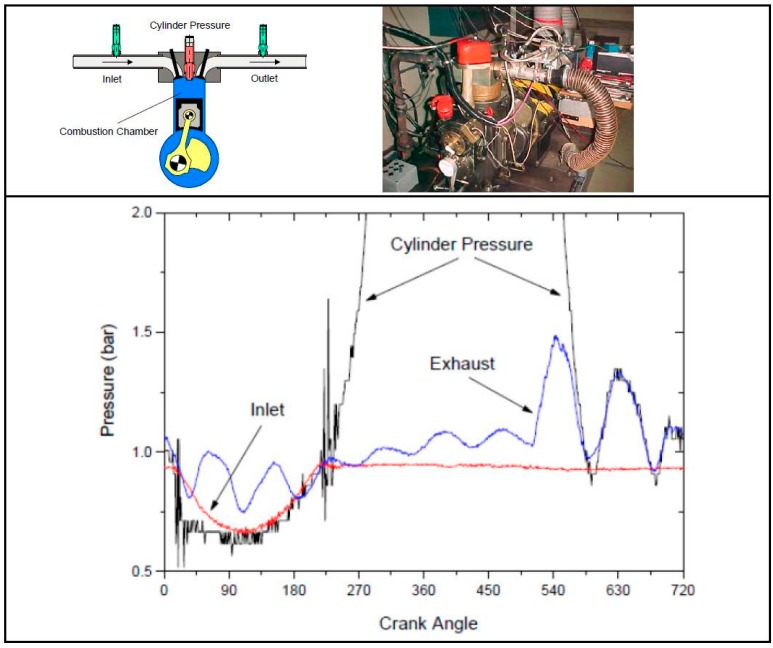
Test results with the fabricated sensor as inlet and exhaust sensor in combustion engine in compare with reference sensor [9].

The packaged sensor was tested in inlet and exhaust manifolds of a combustion engine. Figure 13 shows the operation and the dynamic pressure signals of an internal combustion engine operated at 1500 rpm under full load. The signals are compared with the cylinder pressure measured in the combustion chamber. The sensor shows much better resolution and very good and reliable sensor behavior.

## 5. Conclusions

Two comprehensive sensor packaging concepts and the fabrication of sensor elements for use in harsh environments were presented. In both concepts transfer media like Hg, oil or NaK, which are expensive and require complicated filling technologies are no longer needed. In the case of the flip-chip-sensor concept the sensor chip is mounted using flip-chip technology to avoid the use of wire bonding technology. Gluing is not necessary. The pressure range can be adjusted by the steel membrane thickness. The fabricated SOI-based sensors with this packaging concept have been successfully tested in combustion engines and plastic injection molding equipment and have shown excellent behavior.

The presented “floating” sensor concept avoids the use of any additional materials, such as needed in common chip bonding technologies, so the high stress resulting from the different thermal expansion coefficients of different materials can be reduced. The sensor systems have shown very stable and repeatable behavior. No visible damage has been observed after long term tests [10]. 
